# Mediational Patterns of Parenting Styles Between Cognitive Disengagement Syndrome Difficulties and Youth Psychopathology

**DOI:** 10.3390/children12091134

**Published:** 2025-08-27

**Authors:** Ludovica Giani, Stefano De Francesco, Cecilia Amico, Gaia De Giuli, Marcella Caputi, Simona Scaini

**Affiliations:** 1 Child & Youth Lab, Sigmund Freud University of Milan, Via Ripa di Porta Ticinese 77, 20143 Milan, Italy; l.giani@milano-sfu.it (L.G.);; 2 Dipartimento di Scienze della Vita, University of Trieste, Via E. Weiss, 2, 34128 Trieste, Italy; 3 Cliniche Italiane di Psicoterapia Età Evolutiva, Gruppo Studi Cognitivi, Corso San Gottardo 5, 20143 Milan, Italy

**Keywords:** cognitive disengagement syndrome, parenting styles, internalizing and externalizing symptoms, children

## Abstract

**Highlights:**

**What are the main findings?**

- Negative parenting practices (poor monitoring, inconsistent discipline, corporal punishment) partially mediate the relationship between Cognitive Disengagement Syndrome (CDS) symptoms and youth anxiety, depression, and oppositional–defiant symptoms.
- Positive parenting practices, although beneficial, did not play a significant mediating role in these associations.

**What is the implication of the main finding?**

- Children’s CDS-related difficulties may lead parents to adopt negative parenting behaviors, which in turn exacerbate psychopathological symptoms in their offspring.
- Clinical and preventive interventions should prioritize reducing negative parenting practices, alongside promoting positive ones, to mitigate the impact of CDS on youth mental health.

**Abstract:**

Background/Objectives: Cognitive Disengagement Syndrome (CDS) is a clinical condition primarily characterized by inattention, hypoactivity, and mind-wandering, which has not yet been recognized as an official diagnostic category. Although there are overlaps between CDS and ADHD, evidence supports the semi-independence of CDS from the ADHD-Inattentive subtype. Importantly, while the impact of ADHD on parenting styles has been studied, no previous research has investigated the potential influence of CDS difficulties on parenting behaviors. Both CDS and ADHD are associated with internalizing and externalizing symptoms, which are influenced by negative parenting styles. The severity of ADHD is known to predict the use of dysfunctional parenting patterns; however, no studies have yet investigated how CDS difficulties might affect parenting styles. Due to the similarities between CDS and ADHD, it is reasonable to hypothesize a similar relationship. This study aims to examine the potential mediating role of parenting styles—both negative and positive—in the relationship between CDS difficulties and internalizing and externalizing symptoms. Methods: The sample is composed of 369 Italian school-aged children (9.38 ± 2.34 years old). Parents reported on their children’s psychopathology, CDS difficulties, and their own parenting strategies. Results: Analyses conducted using Hayes’ PROCESS tool indicated that only negative parenting styles partially mediated the relationship between CDS difficulties and parent-reported youth anxiety, depression, and oppositional defiant disorder. Conclusions: These findings highlight the importance of interventions aimed at both addressing CDS in children and improving parenting strategies to enhance youth psychopathological outcomes.

## 1. Introduction

Cognitive Disengagement Syndrome (CDS), previously known as Sluggish Cognitive Tempo (SCT), has been defined by an International Work Group of 13 active leading experts in the field of SCT as a set of developmentally inappropriate caregiver-reported behaviors and symptoms involving slowed-down cognitive processing speed, excessive daydreaming and mind-wandering, mental confusion/fogginess, difficulty initiating and sustaining effort, low motivation, drowsiness, and marked hypoactivity that can impair a child’s daily functioning in several domains [1,2]. The presence of attentional deficits and task-unrelated thoughts referring to the cognitive distinctive dimension of CDS has often led professionals to inappropriately diagnose ADHD inattentive subtype (ADHD-I). The growing body of studies carried out to further explore the nature of this syndrome, that exists only as a research entity but not in official diagnostic taxonomies, has provided mixed findings about its nosography. On one hand, studies have recognized that SCT constitutes a distinct disorder from ADHD either in children [3,4] or adults [1] and from daytime sleepiness, anxiety and depression [5,6,7,8,9] whereas on the other hand, there is also evidence suggesting that SCT might act as a higher order transdiagnostic process that meaningfully predicts risk and functional impairments across psychopathologies [10,11]. The best supported evidence suggests that CDS is a semi-independent set of symptoms from the ADHD as the two clinical conditions may coexist in 39% to 59% of cases [12] due to some overlapping symptoms that make harder differential diagnosis [13,14]. Specifically, a recent study carried out on a sample of American children showed that more than half (59%) of youth who encountered CDS difficulties also fulfilled diagnostic criteria for ADHD, respectively, 22% for ADHD-Inattentive subtype, 8% for ADHD-Hyperactive subtype, and 30% for ADHD-Combined subtype [12]. Considering the reverse relationship, among those who qualified for ADHD, 39% also qualified for CDS [12]. Therefore, such overlaps indicate that CDS and ADHD are distinct but also partially dependent on each other. Nowadays, CDS represents a relevant clinical challenge in terms of diagnostic assessment and clinical interventions due to the well-established impairments that CDS symptoms cause in the academic [2,15,16], social [17,18,19,20], executive [21,22,23], and emotional [24,25] functioning of children above and beyond ADHD symptoms.

The growing literature about CDS supports a greater association of this condition with internalizing disorders than externalizing ones [2,26,27,28].

Children with CDS symptoms may also be more prone to experiencing feelings of sadness or anxiety, as well as low self-esteem. The link with enhanced internalizing symptoms, emotion dysregulation, higher patterns of social withdrawal, isolation, increased punishment sensitivity and a major tendency toward engagement in negative and repetitive style thinking is well-corroborated and might worsen the child’s socio-emotional wellbeing with repercussions on education [27,28,29]. The study by [30] examined the direction of the association between CDS symptoms and anxious/depressive symptoms among school-aged children. Findings suggest that CDS symptoms may confer risk for subsequent depressive symptoms rather than the reverse, whereas findings concerning anxiety are more nuanced and differ according to the rater.

This syndrome is also uniquely associated with greater self-reported mind-wandering and rumination [29], acting like potential mechanisms linking CDS to withdrawal behaviors [31,32], that in turn could maintain depression and disengage children from social supportive relationships. Similarly, [27] showed that children with CDS may have difficulties with assertiveness displaying over-engagement in inner/mental stimuli instead of external events foster isolation, not due to peer rejection as often encountered in ADHD [18,33]. Consequently, struggling with task-unrelated thoughts not only impairs the learning processes at school but also hinders educational and cooperative peer exchanges.

With regard to externalizing symptoms, more contradictory results emerged. Indeed, on one hand, studies highlight that CDS is either positively or negatively associated with hyperactivity, oppositionality, provocativeness, conduct problems, antisocial behaviors and substance use, on the other hand this association is not supported by other evidence when controlling for ADHD-Inattention [7,34,35,36,37]. Particularly, the study by [38] was carried out on a sample of 413 children aged between 6 and 11 years old and outlined that oppositional and defiant disorder as well as conduct disorder appear among the most prevalent comorbidities encountered in youth with CDS. Specifically, externalizing symptoms account for 22.6% of the co-occurring psychopathologies compared to the 29.5% of ADHD-Inattentive subtype and the 31.3% of anxiety disorders. Conversely, a recent study conducted by [2] supported a slight association between CDS and externalizing behaviors that tend to become non-significant or even negative when co-varying ADHD symptoms [10].

Given the high comorbidity rate between CDS and Internalizing and Externalizing symptoms, recent studies focused on the detection of possible common etiopathological factors at the basis of this co-occurrence. Notably, a cutting-edge twin study by [39] outlined that genetic factors only partially account for the covariance of CDS and somatic anxiety as well as generalized anxiety disorder, being instead the unique environment the main responsible for it. However, it still has to be clarified which non-shared environmental factor plays a pivotal role in this association. A growing body of literature conceptualized parenting as one of the most important non-shared environmental influences on problem behaviors of offsprings [40,41,42,43]. Parenting styles refer to general patterns of childrearing, influenced by the temperament of both individuals engaged in the relationship, that characterize parents’ typical responses to their children’s requests [44]. Notably, [45] showed that negative parenting behaviors (e.g., negative communication and harsh control) are positively linked to internalizing symptoms and, conversely, positive parenting behaviors (e.g., parental warmth, monitoring) are inversely related to internalizing disorders. Likewise, the meta-analysis by [46] identified moderate positive associations between negative parenting and externalizing symptoms and only weak links with the same psychopathological dimension for positive parenting behaviors.

Moreover, there is extensive evidence supporting the assumption that parenting styles might be significantly influenced by offsprings’ behavioral problems. Specifically, having a child with ADHD poses strong challenges to the family dynamics as the effect of this neurodevelopmental condition are not limited to the child alone but affect their closest social contexts, including family too [47,48]. Although bidirectional influences exist between ADHD and parenting styles, so that child’s negative behaviors increase the likelihood of parents to adopt harsh and negative discipline styles, which in turn, maladaptively reinforce the child’s conduct problems, many researchers found that the severity of ADHD predicts recurring to dysfunctional parenting patterns, such as permissiveness or emotional overreaction [49,50,51,52].

Conversely, no efforts have been made so far to investigate how CDS difficulties might affect parenting styles. However, due to the overlaps between CDS and ADHD, there are sufficient elements to suppose a similar relationship.

In light of these premises, the main objective of the present study is to test the potential mediational role of parenting styles, either negative or positive dimensions, in the relationship between cognitive disengagement syndrome difficulties and internalizing and externalizing symptoms in school-aged children.

## 2. Materials and Methods

### 2.1. Participants and Study Design

The present study has a cross-sectional research design and involves a sample, recruited through a convenience sampling strategy, composed of parents (41.87 ± 5.67 years old, age range: 27–57 years old, 271 mothers and 67 fathers, 31 missing parental gender data) of 369 children (9.38 ± 2.34 years old, age range: 6–14 years old, 181 females and 183 males, 5 missing gender data) attending the first (N = 49), second (N = 41), third (N = 53), fourth (N = 41), and fifth (N = 51) year of the primary school, and the first (N = 32), second (N = 41), and third year (N = 45) of the middle school. The class attendance of 16 children was missing. Most of the recruited children were Italian (N = 185), 106 participants had a foreign citizenship (N = 106), whereas the remaining ones (N = 78) did not indicate their nationality. With regard to the socio-economic status, the sample is slightly heterogenous as 10 participants self-reported an annual income lower than 10.000 euros, 62 respondents declared an annual income ranging from 10.000 to 20.000 euros, 153 between 20.000 and 40.000 euros, 69 between 40.000 and 80.000 euros, whereas only 3 subjects indicated an annual income ranging from 80.000 and 100.000 euros, and other 3 subjects reported an annual income higher than 100.000 euros.

### 2.2. Procedures

Firstly, 30 primary and middle schools based in the North of Italy, in particular in Milan and Mantova, were selected through a convenience recruitment strategy. The principal of each school was contacted via email to broadly present the research project and to assess the willing of the school to participate. Only one school based in Mantova and composed of 14 different departments, namely 5 kindergartens, 6 primary schools and 3 middle schools, granted availability to conduct the research. After initial declaration of interests, an in-person meeting was scheduled with the principal and teachers to present the main objectives of the study, as well as the rationale, methodology, procedures of data collection and analyses. Secondly, the same procedure was replicated with parents of children aged between 6 and 14 years old. Only those who returned signed written informed consent were formally enrolled in the study. Parents of children with Intellectual Disability or Autism Spectrum Disorder were excluded from the study. The initial sample was composed of 397 children, of which 18 later decided not to return the questionnaires and 10 were eliminated due to the lack of the participation approval of both parents. Therefore, the final sample reached 369 subjects. The test battery designed to be completed by parents was given to children with specific instructions to have their parents fill it out. Italian children who were attending primary and middle school, or being aged between 6 and 14 years old, and whose parents did not have comprehensive Italian language difficulties, were selected in the study as fulfilling eligibility criteria for inclusion. Conversely, children whose parents did not know the Italian language were excluded. Data collection was initiated subsequent to the Ethics Committee’s approval of the study on 4 December 2022.

### 2.3. Instruments

Alabama Parenting Questionnaire (APQ [53]) is a self-report measure of parenting practices designed to be filled by caregivers that was used to assess the mediating variables of the present study. It consists of 42 items, whose score ranges on a 5-point Likert scale from 1 (never) to 5 (always), that indicate how frequently the parent employs each specific behavior to regulate the child’s actions in ordinary interactions. The total raw score ranges from a minimum of 42 to a maximum of 210. The APQ yields five subscales, namely: Parental Involvement (PI), Positive Parenting (PP), Poor Monitoring/Supervision (PM), Inconsistent Discipline (ID), and Corporal Punishment (CP). The PI and PP subscales have a positive direction, therefore higher scores indicate positive practices, whereas the PM, ID and CP subscales pursue a negative direction where higher scores reflect ineffective parenting [54]. In the present study, we relied on the dichotomized clustering of the five initial subscales emerged from principal component analysis, considering positive (PI + PP) and negative parenting (PM + ID + CP) as mediators. The APQ Italian version also boasts good psychometric properties, with internal consistency ranging from α = 0.50 (corporal punishment subscale) to α = 0.80 (parental involvement subscale) [53].

Italian Cognitive Disengagement Syndrome Scale (ICDSS: currently under review) is a parent-report instrument aimed at fostering a clinical screening of CDS symptoms in children aged between 6 and 14 years old. In the present study, it was used to assess the independent variable. It consists of 29 items ranging on a 4-point Likert scale from 0 (never/rarely) to 3 (very often/frequently). The total raw score ranges from a minimum of 0 to a maximum of 87. Items are grouped in 3 subscales: “Attention and working memory impairments”, “Hypoactivity and low energy”, “Daydreaming and mental fogginess”. The Italian validation study reported a sufficient-good test–retest reliability ranging from rho = 0.55 to rho = 0.81 and a good-excellent internal consistency that varies from α = 0.83 to α = 0.92 among the three principal components (currently under review).

Child and Adolescent Behavior Inventory (CABI: Refs. [55,56] a parent-report questionnaire composed of 67 items with response options on a 6-point Likert scale (almost never, rarely, sometimes, often, very often, almost always) that investigates emotional and/or behavioral problems in children and adolescents aged between 6 and 17 years old. In the present study, it was used to assess the dependent variables. The tool allows exploration of the following areas: Sluggish Cognitive Tempo symptoms (16 items, total raw score ranges from a minimum of 0 to a maximum of 80), anxiety symptoms (6 items, total raw score ranges from a minimum of 0 to a maximum of 30), depressive symptoms (7 items, total raw score ranges from a minimum of 0 to a maximum of 35), ADHD-Inattention subtype symptoms (9 items, total raw score ranges from a minimum of 0 to a maximum of 45), ADHD-Impulsive and Hyperactive subtype (9 items, total raw score ranges from a minimum of 0 to a maximum of 45), oppositional and defiant symptoms (8 items, total raw score ranges from a minimum of 0 to a maximum of 40), insensibility traits (4 items, total raw score ranges from a minimum of 0 to a maximum of 20), social impairments (4 items, total raw score ranges from a minimum of 0 to a maximum of 24), and academic impairments (4 items, total raw score ranges from a minimum of 0 to a maximum of 24). The Italian validation study reported a good internal consistency among items, as the Cronbach’s alpha indices were 0.82 for the internalizing scale, 0.87 for the externalizing scale, and 87 for ADHD [56].

### 2.4. Ethics

The study was performed in accordance with the Declaration of Helsinki (1975, revised in 2013) and approved by the Ethics Committee of the Sigmund Freud University on 4th December 2022 (protocol n. NCKSH2NMBJR1M89798). Written informed consent was obtained from parents/legal tutors of children at the beginning of the study, following a detailed explanation of the objectives, as well as of the methodological procedures undertaken and data management.

### 2.5. Data Analysis

Descriptive statistics and frequency analyses were carried out for all participants to delineate the main characteristics of the children involved in the study.

Prior to conducting preliminary and main analyses, the form indexes of skewness and kurtosis were analyzed to assess the fulfillment of criteria for determining the normal distribution of the variables of interest, namely Positive APQ, Negative APQ, ICDSS Tot, CABI Anxiety, CABI Depression, CABI ODD. Given that the sample size was superior to 300 subjects, absolute skewness values ≤ 2 or absolute kurtosis values ≤ 4 were considered as reference values for determining normality [57]. In light of these premises, all variables that were not normally distributed were adjusted using the logarithmic function Log_10_(x + 2), as mediation models rely on linear regressions analyses, for which the assumption of normality represents a fundamental prerequisite. A comparison of mean analysis, specifically a series of independent samples *t*-tests were carried out in order to exclude any potential gender differences on the main youth psychopathological dimensions investigated, namely CDS, anxiety, depressive and oppositional and defiant symptoms. Pearson’s bivariate correlations with Bonferroni correction for multiple comparisons were performed among all the study variables to verify potential multicollinearity issues prior to running the mediation models. Finally, three separate mediation models were run to outline the role of positive and negative parenting in the relationship between parent-reported CDS symptoms and offsprings’ symptoms belonging to the internalizing and externalizing domain, under the assumption that mediation can be defined when the association between the independent and dependent variable is at least partially accounted for by the mediating variables. An alpha level of 0.05 (two-tailed) was adopted as the criterion for statistical significance. Statistical analyses were carried out using SPSS 27 [58] and R Studio (Version 2024.09.1+394) [59]. SPSS 27 was implemented to carry out all the inferential statistics, whereas R Studio was used to perform the mediation analysis using the Laavan package.

## 3. Results

### 3.1. Preliminary Analyses

The independent-samples *t*-tests highlighted no significant gender differences in the main youth psychopathological dimensions investigated, namely CDS (t(307) = 0.50, *p* = 0.31), anxiety (t(310) = −0.62, *p* = 0.27), depressive (t(326) = −1.10, *p* = 0.14), and oppositional and defiant (t(326) = 0.65, *p* = 0.26) symptoms. Therefore, gender was not used in the mediation models as moderator. Moreover, before commenting on the main findings, a series of Pearson’s bivariate correlations with Bonferroni correction for multiple comparisons were performed among all the study variables and results are shown in Table 1.

### 3.2. Main Analyses: Mediation Model

Following our data analysis plan, three separate mediation models, described by [60] as multiple model 4 of mediation, were tested. In the first model, parent-reported Cognitive Disengagement Syndrome’s symptoms of their offsprings, represented by the ICDSS total score, was the independent variable (X), parent-reported anxiety symptoms, represented by the score of the CABI Anxiety subscale, was the dependent variable (Y), and Positive (subscale 1 and 2 of APQ) and Negative Parenting (subscale 3, 4 and 5 of APQ) embodied the moderators (M1 and M2, respectively). In the second and third model, the same mediation model was carried out replacing the outcome measure with the Depressive and Oppositional and Defiant Behaviors, represented by the CABI related subscales.

#### 3.2.1. First Mediation Model

**Figure 1:**
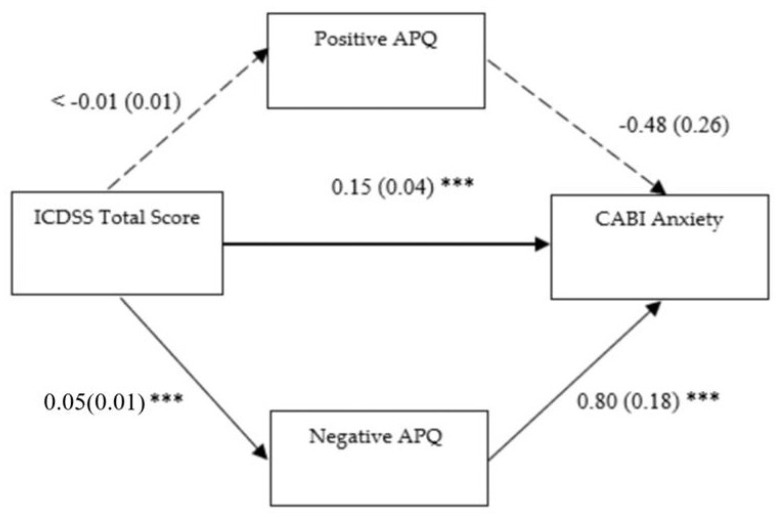
Positive and negative parenting as mediator of the association between parent-reported CDS symptoms and anxiety ones. **Notes:** ICDSS: Italian Cognitive Disengagement Syndrome Scale; Positive APQ: “Involvement” + “Positive Parenting” subscales of the Alabama Parenting Questionnaire; Negative APQ: “Poor Monitoring/Supervision” + “Inconsistent Discipline” + “Corporal Punishment” subscales of the Alabama Parenting Questionnaire; CABI Anxiety: Anxiety subscale of the Child and Adolescent Behavior Inventory; Unstandardized coefficients are reported with standard errors in parentheses. Analyses were based on 1000 bootstrap samples with 95% bias-corrected confidence intervals; *** *p* ˂0.001.

In the first model (see Figure 1), all the direct paths were significant, except for the direct relationship between parent-reported CDS symptoms and positive parenting (ß = −0.004, SE = 0.01, *p* = 0.60, 95% CI [−0.02, 0.01]) as well as positive parenting and youth anxiety symptoms (ß = −0.48, SE = 0.26, *p* = 0.07, 95% CI [−1.04, −0.01]). Notably, parent-reported CDS symptoms had a statistically significant effect on negative parenting (ß = 0.05, SE = 0.01, *p* ≤ 0.001, 95% CI [0.03, 0.07]), as well as on youth anxiety symptoms (ß = 0.15, SE = 0.04, *p* ≤ 0.001, 95% CI [0.08, 0.23]); whereas negative parenting showed a relevant direct path with youth anxiety symptoms (ß = 0.80, SE = 0.18, *p* ≤ 0.001, 95% CI [0.45, 1.16]). The total effect of the predictor on the outcome via M1 was significant (ß = 0.15, SE = 0.04, *p* ≤ 0.001, 95% CI [0.08, 0.23]) net of an indirect effect on the same paths that was not statistically significant (ß = 0.002, SE = 0.004, *p* = 0.66, 95% CI [−0.006, 0.012]). The total effect of the predictor on the outcome via M2 was statistically significant (ß = 0.19, SE = 0.04, *p* ≤ 0.001, 95% CI [0.12, 0.26]) as well as the indirect effect on the same paths (ß = 0.04, SE = 0.01, *p* = 0.002, 95% CI [0.02, 0.07]). Therefore, negative parenting practices partially mediate the relationship between parent-reported CDS symptoms and youth anxiety symptoms; whereas positive parenting practices do not act as mediators in the same relationship.

#### 3.2.2. Second Mediation Model

**Figure 2:**
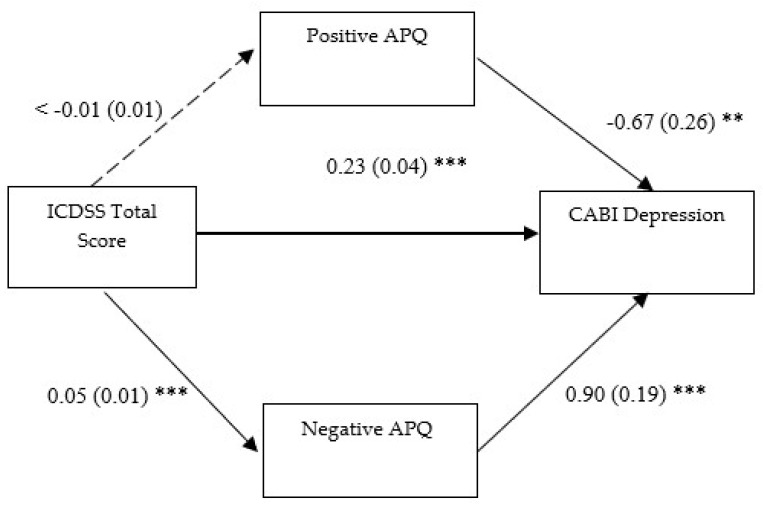
Positive and negative parenting as mediator of the association between parent-reported CDS symptoms and depressive ones. Notes: ICDSS: Italian Cognitive Disengagement Syndrome Scale; Positive APQ: “Involvement” + “Positive Parenting” subscales of the Alabama Parenting Questionnaire; Negative APQ: “Poor Monitoring/Supervision” + “Inconsistent Discipline” + “Corporal Punishment” subscales of the Alabama Parenting Questionnaire; CABI Depression: Depression subscale of the Child and Adolescent Behavior Inventory; Unstandardized coefficients are reported with standard errors in parentheses. Analyses were based on 1000 bootstrap samples with 95% bias-corrected confidence intervals; ** *p* ˂ 0.01; *** *p* ˂ 0.001.

In the second model (see Figure 2), all the direct paths were significant, except for the direct relationship between parent-reported CDS symptoms and positive parenting (ß = −0.004, SE = 0.01, *p* = 0.60, 95% CI [−0.02, 0.01]). Notably, parent-reported CDS symptoms had a statistically significant effect on negative (ß = 0.05, SE = 0.01, *p* ≤ 0.001, 95% CI [0.03, 0.07]) parenting, as well as on youth depressive symptoms (ß = 0.23, SE = 0.04, *p* ≤ 0.001, 95% CI [0.15, 0.30]); whereas either positive (ß = −0.67, SE = 0.26, *p* = 0.01, 95% CI [−1.17, −0.12]) or negative (ß = 0.90, SE = 0.19, *p* ≤ 0.001, 95% CI [0.56, 1.33]) parenting showed a relevant direct path with youth depressive symptoms. The total effect of the predictor on the outcome via M1 was significant (ß = 0.23, SE = 0.04, *p* ≤ 0.001, 95% CI [0.15, 0.30]) net of an indirect effect on the same paths that was not statistically significant (ß = 0.003, SE = 0.01, *p* = 0.63, 95% CI [−0.01, 0.02]). The total effect of the predictor on the outcome via M2 was statistically significant (ß = 0.27, SE = 0.04, *p* ≤ 0.001, 95% CI [0.20, 0.34]), as well as the indirect effect on the same paths (ß = 0.04, SE = 0.01, *p* = 0.001, 95% CI [0.02, 0.07]). Therefore, negative parenting practices partially mediate the relationship between parent-reported CDS symptoms and youth depressive symptoms whereas positive parenting practices do not act as mediators in the same relationship.

#### 3.2.3. Third Mediation Model

**Figure 3:**
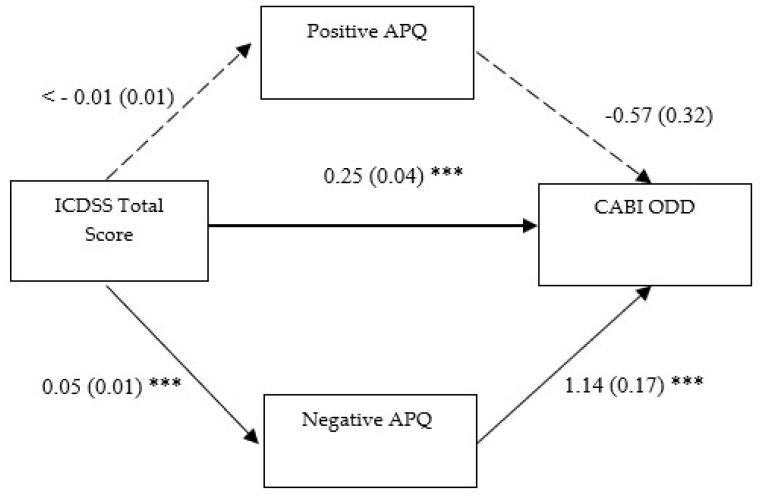
Positive and negative parenting as mediator of the association between parent-reported CDS symptoms and oppositional and defiant ones. Notes: ICDSS: Italian Cognitive Disengagement Syndrome Scale; Positive APQ: “Involvement” + “Positive Parenting” subscales of the Alabama Parenting Questionnaire; Negative APQ: “Poor Monitoring/Supervision” + “Inconsistent Discipline” + “Corporal Punishment” subscales of the Alabama Parenting Questionnaire; CABI ODD: Oppositional and Defiant Disorder subscale of the Child and Adolescent Behavior Inventory; Unstandardized coefficients are reported with standard errors in parentheses. Analyses were based on 1000 bootstrap samples with 95% bias-corrected confidence intervals; *** *p* ˂ 0.001.

In the third model (see Figure 3), all the direct paths were significant, except for the direct relationship between parent-reported CDS symptoms and positive parenting (ß = −0.004, SE = 0.01, *p* = 0.60, 95% CI [−0.02, 0.01]) as well as positive parenting and youth oppositional and defiant symptoms (ß = −0.57, SE = 0.32, *p* = 0.07, 95% CI [−1.25, 0.02]). Notably, parent-reported CDS symptoms had a statistically significant effect on negative (ß = 0.05, SE = 0.01, *p* ≤ 0.001, 95% CI [0.03, 0.07]) parenting, as well as on youth oppositional and defiant symptoms (ß = 0.25, SE = 0.04, *p* ≤ 0.001, 95% CI [0.18, 0.32]); whereas negative parenting show a relevant direct path with youth oppositional and defiant symptoms (ß = 1.51, SE = 0.17, *p* ≤ 0.001, 95% CI [1.20, 1.87]). The total effect of the predictor on the outcome via M1 was significant (ß = 0.26, SE = 0.04, *p* ≤ 0.001, 95% CI [0.18, 0.32]) net of an indirect effect on the same paths that was not statistically significant (ß = 0.002, SE = 0.01, *p* = 0.63, 95% CI [−0.01, 0.01]). The total effect of the predictor on the outcome via M2 was statistically significant (ß = 0.33, SE = 0.04, *p* ≤ 0.001, 95% CI [0.25, 0.40]) as well as the indirect effect on the same paths (ß = 0.07, SE = 0.02, *p* ≤ 0.001, 95% CI [0.04, 0.11]). Therefore, negative parenting practices partially mediate the relationship between parent-reported CDS symptoms and youth oppositional and defiant symptoms; whereas positive parenting practices do not act as mediators in the same relationship.

In other words, having an offspring who reports a high score on the ICDSS might yield parents to adopt negative behaviors, such as poor monitoring and supervision, inconsistent discipline and corporal punishment towards their children that, in turn, contributes to heightening youth anxiety, depressive and oppositional and defiant symptoms.

## 4. Discussion

This study aimed to explore the mediating role of positive and negative parenting practices in the relationship between parent-reported CDS symptoms and various youth psychopathological outcomes, specifically anxiety, depressive, and oppositional and defiant symptoms. Given the increasing recognition of CDS and its impact on youth mental health, understanding if and how parental behaviors mediate this relationship is crucial for developing targeted interventions. By investigating these mediation effects, the study sought to identify specific parenting practices that could mitigate or exacerbate the adverse effects of CDS symptoms on youth psychopathology.

The preliminary analyses indicated no significant gender differences in the primary psychopathological dimensions investigated, including CDS, anxiety, depressive, and oppositional and defiant symptoms. This result aligns with some prior studies but diverges from others that identified significant gender effects in youth psychopathology [61]. The absence of gender differences allowed us to exclude gender as a moderator in the mediation models, simplifying the analysis and focusing on the primary variables of interest. Additionally, Pearson’s bivariate correlations, adjusted with Bonferroni correction, provided a comprehensive overview of the relationships among all study variables, which informed the subsequent mediation analyses.

The main analyses explored the mediating role of positive and negative parenting practices in the relationship between parent-reported CDS symptoms and various youth psychopathological outcomes through three separate mediation models. In the first model, negative parenting practices partially mediated the relationship between parent-reported CDS symptoms and youth anxiety symptoms, whereas positive parenting practices did not act as significant mediators [61]. Specifically, CDS symptoms had a significant direct effect on negative parenting, as well as on youth anxiety symptoms. Negative parenting also exhibited a significant direct effect on youth anxiety symptoms. These findings suggest that CDS symptoms in youth may prompt parents to engage in negative parenting behaviors such as poor monitoring and inconsistent discipline, which in turn exacerbate anxiety symptoms in youth. The lack of mediation by positive parenting implies that although supportive and nurturing behaviors are beneficial, they might not substantially mitigate the negative effects of CDS symptoms and negative parenting on youth anxiety. These findings align with those reported by [62], which indicate that negative parenting styles—characterized by children’s perception of excessive control and strict discipline—are associated with higher levels of anxiety compared to positive parenting styles, which encourage independence while maintaining appropriate limits and behavioral control.

The second model demonstrated a similar pattern. Negative parenting practices again partially mediated the relationship between CDS symptoms and youth depressive symptoms, while positive parenting practices did not. Parent-reported CDS symptoms significantly influenced both positive and negative parenting practices, as well as youth depressive symptoms, with negative parenting showing a robust direct effect on depressive symptoms. This highlights the harmful impact of negative parenting on youth depression, emphasizing the need to address negative parenting behaviors in therapeutic interventions. The non-significant mediating role of positive parenting suggests that its beneficial effects may be insufficient to counterbalance the adverse effects of negative parenting behaviors on youth depression. Our findings align with a growing body of research showing that negative parenting practices exert a stronger influence on youth depressive symptoms than positive parenting practices. For instance, ref. [63] conducted a meta-analysis demonstrating that parental rejection, hostility, and psychological control are robust predictors of child and adolescent depression, whereas parental warmth and support show weaker or inconsistent protective effects. Similarly, ref. [64], in a systematic review, identified low parental warmth and high levels of control and criticism as consistent risk factors for the development of depressive and anxiety symptoms in young people. More recent longitudinal studies also confirm that negative parenting behaviors such as harsh discipline and overcontrol are associated with increased depressive symptoms in adolescents, while positive parenting practices, although beneficial, often fail to buffer against the detrimental impact of negative behaviors [65]. These converging findings reinforce the interpretation that interventions aiming to prevent or reduce youth depression should prioritize the reduction in negative parenting practices, while still supporting the enhancement of positive parenting.

In the third model, negative parenting practices partially mediated the relationship between CDS symptoms and oppositional and defiant symptoms, whereas positive parenting practices did not. The direct paths from CDS symptoms to both positive and negative parenting, and to youth oppositional and defiant symptoms, were significant. Negative parenting had a particularly strong direct effect on these behavioral issues, indicating that negative parental behaviors are crucial factors in the development and persistence of oppositional and defiant behaviors in youth. These results are consistent with prior studies showing that harsh, inconsistent, and coercive parenting are among the strongest predictors of oppositional defiant disorder (ODD) and conduct-related problems in children and adolescents. For example, a meta-analysis by [46] demonstrated that high levels of harsh control and low parental warmth are reliably associated with externalizing symptoms, including oppositional and defiant behaviors. Similarly, ref. [66] highlighted that negative parenting practices, such as harsh discipline and lack of sensitivity, are robust predictors of callous–unemotional traits and oppositional behavior. Moreover, longitudinal work by [67] emphasized that reductions in harsh and inconsistent parenting are linked to decreases in externalizing symptoms over time.

Taken together, these findings suggest that interventions targeting these behaviors should prioritize reducing negative parenting practices to mitigate their impact on youth.

The consistent pattern observed across all three models highlights the critical mediating role of negative parenting practices in the relationship between parent-reported CDS symptoms and various youth psychopathological outcomes. Parenting can therefore constitute a possible common etiopathological factor underlying this co-occurrence and can explain secondary outcomes in CDS [68]. Thus, the present findings suggest that interventions aimed at reducing youth psychopathology should focus on decreasing negative parenting behaviors, such as poor monitoring, inconsistent discipline, and corporal punishment. Addressing these negative behaviors may alleviate some of the anxiety, depressive, and oppositional and defiant symptoms experienced by youth. Moreover, the lack of significant mediation by positive parenting practices in these models indicates that while positive parenting behaviors are beneficial, they may not be sufficient to counteract the negative impacts of CDS symptoms and negative parenting on youth psychopathology. This underscores the need for a dual approach in interventions: promoting positive parenting behaviors while simultaneously reducing negative ones.

This study underscores the importance of addressing negative parenting practices in the context of youth psychopathology, especially for children with high CDS symptoms. Future research should continue to explore the complex interactions between different types of parenting behaviors and various youth outcomes to develop more effective interventions. Additionally, understanding the factors that contribute to negative parenting practices could provide further insights into how to support parents in fostering healthier environments for their children. These findings contribute to a growing body of literature emphasizing the multifaceted nature of youth psychopathology and the critical role of parenting in shaping these outcomes.

Although these results might pave the way for providing pivotal insights into the impact of endogenous and exogenous factors on youth psychopathology, some limitations should be highlighted. Firstly, the cross-sectional research design does not allow to ensure the longitudinal role of parenting styles on the development of successive youth psychopathology. Secondly, the whole test battery relies on parent-reports which tend to underestimate internalizing symptoms and to overestimate externalizing problems. This reliance on a single informant introduces a risk of shared method variance and reporter bias, as parents experiencing high stress may perceive both their child’s difficulties and their own parenting more negatively. Consequently, associations among variables may be inflated, limiting the generalizability of the findings. Future studies should include multiple informants, such as teachers, clinicians, or child/adolescent self-reports, and adopt multimethod assessment approaches to reduce bias and provide a more accurate representation of child psychopathology and parenting behaviors. Moreover, although a relevant heterogeneity of self-reported socio-economic status exists within the sample, the potential moderating role of this variable on the psychopathological outcome has not been investigated, as it was not the main purpose of this study. However, being aware that socio-economic factors can influence both parenting strategies and internalizing/externalizing symptoms, we encourage future studies to better investigate their role.

## Figures and Tables

**Table 1:** Pearson’s bivariate correlations among all the study variables with Bonferroni correction.

Variables	2	3	4	5	6
1. Positive APQ	−0.29 ***	−0.15 **	−0.13 *	−0.20 ***	−0.19 ***
2. Negative APQ	-	0.32 ***	0.31 ***	0.37 ***	0.47 ***
3. ICDSS Tot		-	0.46 ***	0.60 ***	0.54 ***
4. CABI Anxiety			-	0.63 ***	0.49 ***
5. CABI Depression				-	0.55 ***
6. CABI ODD					-

Notes. Positive APQ: “Involvement” + “Positive Parenting” subscales of the Alabama Parenting Questionnaire; Negative APQ: “Poor Monitoring/Supervision” + “Inconsistent Discipline” + “Corporal Punishment” subscales of the Alabama Parenting Questionnaire; ICDSS: Italian Cognitive Disengagement Syndrome Scale; CABI Anxiety: Anxiety subscale of the Child and Adolescent Behavior Inventory; CABI Depression: Depression subscale of the Child and Adolescent Behavior Inventory; CABI ODD: Oppositional and Defiant Disorder subscale of the Child and Adolescent Behavior Inventory; * *p* ˂ 0.05; ** *p* ˂ 0.01; *** *p* ˂ 0.001.

## Data Availability

Data will be shared upon direct request to the authors of the present study.

## Abbreviations

The following abbreviations are used in this manuscript:

CDS	Cognitive Disengagement Syndrome
APQ	Alabama Parenting Questionnaire
ICDSS	Italian Cognitive Disengagement Syndrome Scale
CABI	Child and Adolescent Behavior Inventory
